# Genetic characterization of type 2a canine parvoviruses from Taiwan reveals the emergence of an Ile324 mutation in VP2

**DOI:** 10.1186/1743-422X-11-39

**Published:** 2014-02-25

**Authors:** Chao-Nan Lin, Chi-Hsien Chien, Ming-Tang Chiou, Ling-Ling Chueh, Meng-Yu Hung, Han-Siang Hsu

**Affiliations:** 1 Department of Veterinary Medicine, National Pingtung University of Science and Technology, Pingtung, Taiwan; 2 Veterinary Hospital, National Pingtung University of Science and Technology, Pingtung, Taiwan; 3 Graduate Institute of Veterinary Medicine, National Taiwan University, Taipei, Taiwan; 4 Don-Da Animal Hospital, Taichung, Taiwan

**Keywords:** Canine parvovirus, Genotype, VP2, Sequence analysis

## Abstract

**Background:**

Canine parvovirus 2 (CPV 2) is a major infectious cause of mortality in puppies. The characteristic symptom of CPV 2 disease is intestinal hemorrhage with severe bloody diarrhea. Soon after CPV was first recognized in the late 1970s, the original virus, CPV 2, was replaced in the canine population by strains carrying minor antigenic variants (termed 2a, 2b, and 2c) of the VP2 gene that could be distinguished using monoclonal antibodies and molecular analyses. Here, we provide an updated molecular characterization of the CPV 2 circulating in Taiwan.

**Methods:**

In this study, 28 isolates of CPV 2 from 144 dogs with suspected CPV infection were obtained from northern, central, and southern Taiwan from 2008 to 2012 and screened by PCR. The 28 isolates were sequenced, and a phylogenetic analysis of the VP2 gene was performed.

**Results:**

Of the 28 Taiwanese CPV 2 isolates, 15 were identified as new CPV 2a, and 13 were identified as new CPV 2b. Compared to the reference CPV 2a, all 15 of the CPV 2a sequences collected in this study contain an Ile324 mutation caused by a TAT to ATT mutation at nucleotides 970–972 of the VP2 gene.

**Conclusion:**

Our VP2 sequence data revealed that both types are currently prevalent CPV 2 field strains circulating in Taiwan, and a unique Ile324 VP2 mutation was found in our Taiwanese CPV 2a isolates and recent Asian isolates. CPV 2c was not observed in this study.

## Background

Canine parvovirus (CPV) enteritis is characterized by intestinal hemorrhage with severe bloody diarrhea [1]. The causative agent, CPV 2, was first identified in the late 1970s [2]. CPV is a non-enveloped, linear, single-stranded DNA virus with a genome of approximately 5 kb, and it belongs to the genus *Parvovirus,* together with feline panleukopenia virus (FPV), mink enteritis virus, raccoon parvovirus, and porcine parvovirus [3]. Indeed, CPV 2 is believed to have originated from FPV [4,5], and various hypotheses for how this may have occurred have been suggested, including direct mutation from FPV and contact between cats and dogs kept as companion animals within the same home [5].

An antigenic variant, CPV 2a, developed within a few years after the emergence of CPV 2 [6,7], and another CPV 2 variant, CPV 2b, began appearing in the canine population in the mid-1980s [8]. In 2000, a new antigenic variant, CPV 2c, was first detected in Italy [9]. New antigenic types of CPV 2 have been found in epidemics worldwide and are replacing the original CPV 2. The antigenic variant CPV 2a shows the following substitutions within the VP2 protein: Met87Leu, Ile101Thr, Ala300Gly, and Asp305Tyr. Furthermore, CPV 2b has been confirmed to contain an additional substitution, Asn426Asp [10,11]. These two variants further evolved into new 2a and 2b types, with substitutions of Ser297Ala, during the 1990s [12]. Antigenic variant CPV 2c was identified with a substitution Asp426Glu [9]. Different antigenic variants of CPV 2 predominate in different countries [12-42].

A retrospective analysis has revealed that the oldest CPV 2c strain was identified in 1996 in Germany [18], and the results from European epidemiological surveys show that CPV 2c is now predominant in Italy, Germany, and Spain and is also widely co-distributed with CPV 2a or CPV 2b in Portugal, France, and Belgium [18,43-47]. Outside of Europe, CPV 2a and 2b isolates are common in the United States [19,41], whereas CPV 2c is more widespread in Uruguay [20,32], Brazil [33], and Argentina [30,48]. Surprisingly, either CPV 2a or CPV 2b is the predominant variant in Asian countries [12,13,15,21,22,24-26,28,29],[35,37,38] and Australia [42], though a few CPV 2c strains have been isolated in India [26]. Interestingly, a new amino acid substitution, Tyr324Ile, was identified in Korea [21,24], China [29], Thailand [28], Uruguay [32], Japan [38], Taiwan [35], and India [37,49].

In Taiwan, as in other Asian countries, variants CPV 2a and 2b have predominated since the first outbreak [13,15,35]. However, few recent studies have included a genetic analysis of Taiwanese CPV 2 strains. Therefore, the aim of this study was to clarify the evolution of CPV 2 isolated from northern, central, and southern Taiwan during the 2008–2012 period.

## Results

### PCR amplification and genotype analysis

**Figure 1:**
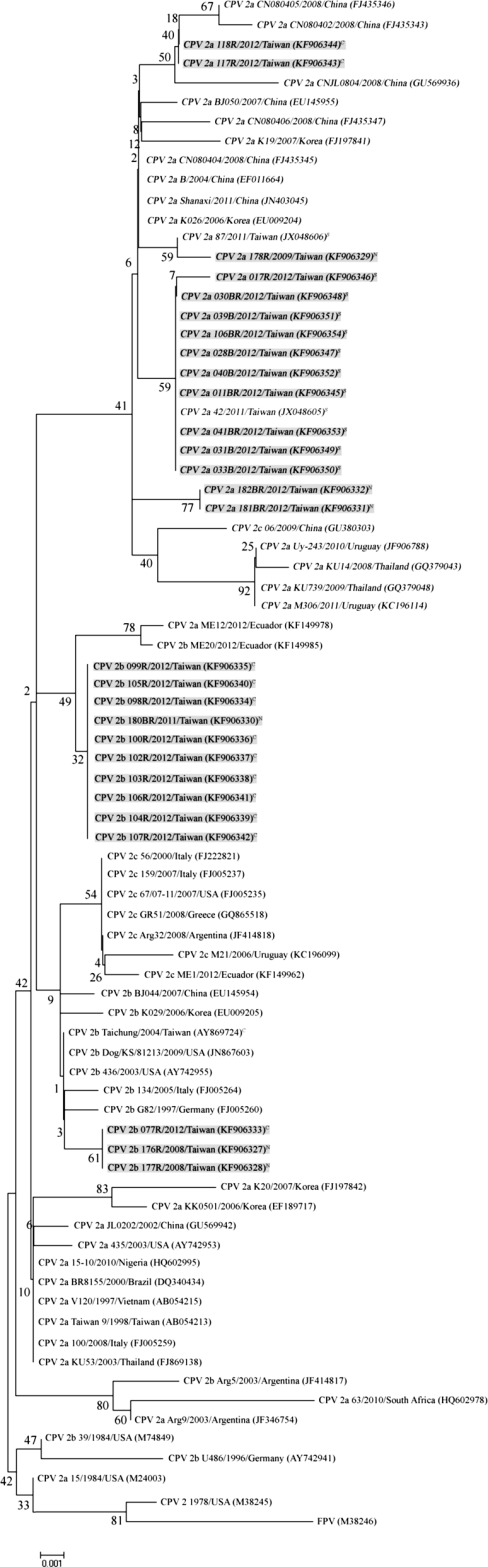
**Phylogenetic relationships based on the partial VP2 gene of CPV 2 between Taiwanese isolates and reference strains.** The analysis was performed employing the maximum likelihood method based on 1,000 replicates using MEGA 5 software. N: Northern Taiwan, C: Central Taiwan, S, Southern Taiwan. Light grey underlay: CPV 2 isolates in the present study. Italic: the Ile324 mutation in VP2.

A total of 28 cases from 144 dogs showed positive results for CPV 2. All of the CPV 2 isolates were clearly separated into two genotypes (Figure 1). Of the 28 Taiwanese CPV 2 strains, 15 were identified as new CPV 2a, and 13 were identified as new CPV 2b. Of the 15 new CPV 2a isolates, 3 (20%), 2 (13.3%), and 10 (66.7%) isolates were collected from northern, central, and southern Taiwan, respectively (Table 1). Of the 13 new CPV 2b isolates, 3 (23.1%), 10 (76.9%), and 0 (0%) isolates were collected from northern, central, and southern Taiwan, respectively (Table 1). Taken together, our results show that both types are currently prevalent CPV 2 field strains circulating in Taiwan.

**Table 1:** The genotypes of 28 canine parvovirus type 2 isolates collected from Taiwanese dogs

Strain^*^	**Sampling time**	**County (Region)**	Age^**^	**Vaccination**	**Genotype**	**Accession number**
176R	2008/JUL	Taipei (North)	NA	NA	2b	KF906327
177R	2008/OCT	Taipei (North)	NA	NA	2b	KF906328
178R	2009/JAN	Taipei (North)	6Y	Yes	2a	KF906329
180BR	2011/DEC	Taipei (North)	NA	NA	2b	KF906330
181BR	2012/JAN	Taipei (North)	4.5 M	No	2a	KF906331
182BR	2012/JAN	Taipei (North)	4.5 M	No	2a	KF906332
077R	2012/JUL	Taichung (Central)	6Y	Yes	2b	KF906333
098R	2012/OCT	Taichung (Central)	3 M	Yes	2b	KF906334
099R	2012/OCT	Taichung (Central)	1Y	Yes	2b	KF906335
100R	2012/OCT	Taichung (Central)	1Y	No	2b	KF906336
102R	2012/NOV	Taichung (Central)	3 M	NA	2b	KF906337
103R	2012/NOV	Taichung (Central)	1Y	No	2b	KF906338
104R	2012/NOV	Taichung (Central)	1Y	No	2b	KF906339
105R	2012/NOV	Taichung (Central)	4 M	No	2b	KF906340
106R	2012/NOV	Taichung (Central)	10 M	No	2b	KF906341
107R	2012/NOV	Taichung (Central)	1Y	No	2b	KF906342
117R	2012/DEC	Taichung (Central)	6 M	No	2a	KF906343
118R	2012/DEC	Taichung (Central)	6 M	No	2a	KF906344
011BR	2012/MAR	Pingtung (South)	2 M	No	2a	KF906345
017R	2012/MAR	Tainan (South)	2 M	Yes	2a	KF906346
028B	2012/MAY	Pingtung (South)	1Y	Yes	2a	KF906347
030BR	2012/MAY	Pingtung (South)	3 M	Yes	2a	KF906348
031B	2012/MAY	Pingtung (South)	4 M	No	2a	KF906349
033B	2012/MAY	Pingtung (South)	1 M	No	2a	KF906350
039B	2012/MAY	Pingtung (South)	3 M	No	2a	KF906351
040B	2012/MAY	Pingtung (South)	3 M	No	2a	KF906352
041BR	2012/MAY	Pingtung (South)	3 M	No	2a	KF906353
106BR	2012/NOV	Pingtung (South)	1Y	Yes	2a	KF906354

^*^The letters in each name indicate the specimen source: B, whole blood; R, rectal swab.

^**^Age of the dog at presentation, Y = years; M = months; NA: not available.

### DNA sequence analysis

The partial VP2 nucleotide sequences were analyzed using DNASTAR software, revealing 99.4-100, 99.7-100, and 99–99.4% homology within the local CPV 2a isolates, within the local CPV 2b isolates, and between the local CPV 2a and 2b isolates, respectively (Table 2). In comparison to the low nucleotide sequence similarity between reference Taiwanese CPV 2a and our CPV 2a (99.4-99.5%), the homology levels between our analyzed CPV 2a isolates (99.7-99.8%) and Korean CPV 2 K026 (EU009204) appeared to be much higher (data not shown).

**Table 2:** Sequence homology of local new CPV 2a and 2b isolates and reference strains

**Strains**	**Genotypes**					
	**New CPV 2a**			**New CPV 2b**		
	**AY742953 (CPV 2a-435)**	**AB054213 (Taiwan 9)**	Taiwanese CPV 2a^*^	**AY742955 (CPV 2b-436)**	**AY869724 (Taichung)**	Taiwanese CPV 2b^**^
AY742953 (CPV 2a-435)	100.0	99.8	99.2-99.4	99.7	99.7	99.5
AB054213 (Taiwan 9)		100.0	99.4-99.5	99.8	99.8	99.7
Taiwanese CPV 2a^*^			99.4-100.0	99.2-99.4	99.2-99.4	99.0-99.4
AY742955 (CPV 2b-436)				100.0	100.0	99.8
AY869724 (Taichung)					100.0	99.8
Taiwanese CPV 2b^**^						99.7-100.0

^*^Accession numbers: KF906329, KF906331, KF906332, KF906343, KF906344, KF906345, KF906346, KF906347, KF906348, KF906349, KF906350, KF906351, KF906352, KF906353, KF906354.

^**^Accession numbers: KF906327, KF906328, KF906330, KF906333, KF906334, KF906335, KF906336, KF906337, KF906338, KF906339, KF906340, KF906341, KF906342.

### Amino acid sequence analysis

**Figure 2:**
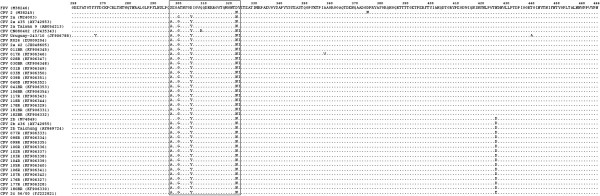
**Multiple amino acid sequence alignment of the partial VP2 gene of CPV 2.** Only those amino acid sequences differing from the FPV sequence are shown. The heterogenic region is boxed.

Amino acid comparisons among the 28 isolates and the 12 reference strains revealed a major region of great diversity at amino acids 297–324 (Figure 2). We also examined the 2 amino acids (positions 297 and 426) within this partial VP2 gene identified by Ohshima [12] et al. and Buonavoglia et al. [9] that were proposed to distinguish CPV 2a/2b from new CPV 2a/2b and CPV 2a/2b/2c, respectively. Our alignment revealed that all of our new CPV 2a and 2b strains contained Ala297 (Figure 2). The amino acid Glu426, which is unique to strain CPV 2c, was no observed in any strain in this study. Four nonsynonymous mutations were observed in our CPV 2a and/or 2b strains (TGT to TGC, CTA to CTG, AAT to AAC and CAA to CAG). One unique amino acid substitution was found in the all of our CPV 2a isolates (Tyr324Ile), caused by the mutation of TAT to ATT at nucleotide positions 970–972 of the VP2 gene. Our CPV 2a isolates were more closely related to the Ile324 isolates from other countries than to the prototype Taiwanese CPV 2a Taiwan 9 (Figure 1).

## Discussion

CPV 2, which causes intestinal hemorrhage with severe bloody diarrhea in dogs, is distributed worldwide, and genetic variation among CPV 2 isolates could be used to further classify the viruses into four genotypes (2, 2a, 2b, and 2c) that differ in their amino acid sequence and VP2 gene phylogenetic relationships [10]. CPV 2a and 2b are the predominant variants in Asia [12,13,15,21,22,24-26,28,29],[35,37,38], whereas CPV 2c is recently distributed on several continents, including Europe (Italy, Germany, Spain, Portugal, France, Belgium, UK, Greece, and Bulgaria) [18,43], Africa (Tunisia) [23], North America (USA) [19,41], South America (Brazil [33], Uruguay [20,32], Argentina [30,48], and Ecuador [34]), and Asia (India) [26]. The first case of CPV 2c was identified in 1996 in Germany [18]. Although a few CPV 2c cases have been reported in Asia (India) [26], no CPV 2c was observed in the present study.

This is the first study to investigate the genotype prevalence of CPV 2 in northern, central, and southern Taiwan in recent years. Our results indicate that both types are currently prevalent CPV 2 field strains circulating in Taiwan. Surprisingly, all of the CPV 2a isolates contain a unique amino acid substitution (Tyr324Ile). Our review of the GenBank database and sequence analyses showed that this Ile324 variant of CPV 2a is also found in Korea [21,24], China [29], Thailand [28], Uruguay [32], Japan [38], Taiwan [35], and India [37,49]. Interestingly, with the exception of Uruguay, this Ile324 CPV 2a variant is only distributed in Asian countries, and our data revealed that this variant was first found in Taiwan in 2009. We believe that the Ile324 CPV 2a variant emerged a few years ago. Taken together, our results suggest that the Ile324 CPV 2a variant may be present due to i) importation from abroad or ii) evolution from existing CPV 2a genotypes. An ongoing investigation is aimed at distinguishing between these possibilities.

VP2 encodes a viral capsid protein that is the major structural protein of CPV 2 and is involved in the host immune response [50]. Therefore, a small number of mutations may result in increased pathogenicity [34], and the effectiveness of commercial vaccines against the Ile324 variant of CPV 2a requires further evaluation. Among all carnivore parvoviruses, residue 324 of VP2 is subject to positive selection [51] and is adjacent to a residue (amino acid 323) known to be involved in host range and tropism via canine transferrin receptor binding [52]. The mutation of CPV 2 residue 323 may influence interactions between residues in neighboring loops of either the same VP2 molecule or the threefold-related VP2, greatly decreasing replication in canine cells [53]. Although the function of residue 324 remains to be elucidated, using SYBR Green-based real-time PCR, our previous study detected the viral shedding of the Ile324 CPV 2 variant for up to 63 days in naturally infected dogs [54]. The pathogenesis of this new variant requires further investigation.

The first CPV 2 infection in Taiwan was recorded in 1980 and was attributed to CPV 2, which was later replaced by CPV 2a and 2b [13]. Previous studies have shown that the predominant genotypes of CPV 2 in northern, central, and southern Taiwan were CPV 2a [13], CPV 2b [15], and CPV 2a [35], respectively, in agreement with our results. Thus, both genotypes (CPV 2a and CPV 2b) constitute the prevalent CPV 2 field strains circulating in Taiwan in the last two decades. However, CPV 2 is constantly mutating, leading to the evolution of novel CPV 2 variants. For example, the CPV 2a Ile324 variant and CPV 2c Glu426 variant have emerged worldwide. Additional CPV 2 cases need to be investigated using continuous surveillance and sequence analysis.

## Conclusion

Our VP2 sequence data revealed that both types are currently prevalent CPV 2 field strains circulating in Taiwan, and a unique Ile324 VP2 mutation was found in our Taiwanese CPV 2a isolates and in recent Asian isolates. CPV 2c was not observed in this study.

## Methods

### Specimen collection and DNA extraction

Clinical samples (whole blood and/or rectal swab) were collected from 144 dogs in northern, central, and southern Taiwan between 2008 and 2012. These samples were mainly acquired from dogs with diarrhea and/or bloody diarrhea. The year of sampling, age, clinical history, and CPV types of the sampled dogs are summarized in Table 1.

### Sample preparation and CPV 2 screening

Viral DNA was extracted from the clinical samples (either whole blood or rectal swab) using a Genomic DNA Mini Kit (Geneaid Biotech, Ltd., Taipei, Taiwan) according to the manufacturer’s protocol. All of the clinical specimens were screened for CPV 2 by polymerase chain reaction (PCR) as described by Lin et al. [54].

### VP2 gene amplification and sequencing

Samples showing positive PCR results from either type of specimen were included in this study. The partial VP2 gene of CPV 2 was amplified by PCR as described by Buonavoglia et al. [9]. The DNA fragments were purified (Geneaid Biotech, Ltd., Taipei, Taiwan), and the target nucleotide sequences were determined in both orientations using an auto-sequencer (ABI 3730XL, Foster City, CA, USA).

### Sequence and phylogenetic analyses

The VP2 DNA sequences of our samples were compared to those of reference FPV (M38246), CPV 2 (M38245), CPV 2a (M24003), CPV 2b (M74849, AY742941), new CPV 2a (AY742953, AB054213, AB054215, DQ340434, EF011664, EF189717, EU009204, EU145955, FJ005259, FJ197841, FJ197842, FJ435343, FJ435345, FJ435346, FJ435347, FJ869138, GQ379043, GQ379048, GU569936, GU569942, HQ602978, HQ602995, JF346754, JF906788, JN403045, JX048605, JX048606, KC196114, KF149978), new CPV 2b (AY742955, AY869724, EU009205, EU145954, FJ005260, FJ005264, JF414817, JN867603, KF149985), and CPV 2c (FJ005235, FJ005237, FJ222821, GQ865518, GU380303, JF414818, KC196099, KF149962). Multiple alignments of the nucleic acid and amino acid sequences were performed with the Clustal W method using the MegAlign program (DNASTAR, Madison, WI, USA). The phylogenetic analyses were conducted by the maximum likelihood method using MEGA 5, version 5.05.

## Abbreviations

CPV: Canine parvovirus; PCR: Polymerase chain reaction.

## Competing interests

The authors declare that they have no competing interests.

## Authors’ contributions

CNL designed and analyzed the experimental data and wrote the manuscript. CHC, MTC, and LLC managed the study, provided materials and reagents, contributed to the interpretation of the data, and co-wrote the manuscript. MYH and HSH contributed to the DNA extraction and PCR. All of the authors read and approved the final manuscript.
